# Early decomposition in visual word recognition: Dissociating morphology, form, and meaning

**DOI:** 10.1080/01690960701588004

**Published:** 2008-03-18

**Authors:** William D. Marslen-Wilson, Mirjana Bozic, Billi Randall

**Affiliations:** MRC Cognition and Brain Sciences Unit, Cambridge, UK; Centre for Speech and Language, University of Cambridge, Cambridge, UK

## Abstract

The role of morphological, semantic, and form-based factors in the early stages of visual word recognition was investigated across different SOAs in a masked priming paradigm, focusing on English derivational morphology. In a first set of experiments, stimulus pairs co-varying in morphological decomposability and in semantic and orthographic relatedness were presented at three SOAs (36, 48, and 72 ms). No effects of orthographic relatedness were found at any SOA. Semantic relatedness did not interact with effects of morphological decomposability, which came through strongly at all SOAs, even for pseudo-suffixed pairs such as *archer-arch*. Derivational morphological effects in masked priming seem to be primarily driven by morphological decomposability at an early stage of visual word recognition, and are independent of semantic factors. A second experiment reversed the order of prime and target (stem-derived rather than derived-stem), and again found that morphological priming did not interact with semantic relatedness. This points to an early segmentation process that is driven by morphological decomposability and not by the structure or content of central lexical representations.

The role of morphological structure in word recognition and lexical access raises important issues about the nature and structure of the language system. A major empirical and theoretical issue is whether morphological factors provide an independent principle for lexical organisation and processing. This is both a methodological question - whether experimental studies involving morphological contrasts can successfully rule out confounds due to possible form and meaning overlaps -  and a theoretical question about lexical representations and their development - whether effects attributable to morphology can be subsumed under the effects of form and meaning, acting either independently or in interaction with each other (e.g., Plaut & Gonnerman, 2000; Rueckl, Mikolinski, Raveh, Miner, & Mars, 1997; Seidenberg & Gonnerman, 2000). Clearly, the possibility of an answer to the second question requires a convincing answer to the first. In English this is complicated by the fact that words that are morphologically related almost always overlap in form, and usually in meaning as well. In the present research, using an incremental masked priming task, we focus on derivational morphology and ask whether there is evidence for a distinct morphological contribution to the processing of written derived words which is not merely the joint product of semantic and orthographic similarity, and, if so, when this information becomes available during the word recognition process.

The advantage of incremental masked priming, where stimulus onset asynchrony (SOA) is varied across conditions, is that it allows us to track the time-course with which different types of information become available during visual word recognition and lexical access. To the extent that morphological, semantic, and orthographic factors exhibit different patterns over time, this provides a tool for determining whether they make separable contributions to the recognition process, and whether this reflects different stages in the process of mapping from orthographic form to lexical meaning. Rastle, Davis, Tyler, and Marslen-Wilson (2000), for example, used three SOAs (43, 72, and 230 ms) to probe priming effects over time for prime-target pairs varying in semantic, morphological, and orthographic relatedness. They found distinctively different patterns at each SOA. Morphologically and semantically related pairs (e.g., *agreement-agree*) primed at all three SOAs. Morphologically related but semantically unrelated pairs (e.g., *apartment-apart*) which typically do not prime when the prime is overt-also showed priming, but only at the shortest SOA (42 ms). Word-pairs that shared just semantic overlap (e.g., *battle-fight*) only primed at the longest SOA (230 ms), when the prime could be consciously perceived. Orthographic effects were absent across all prime duration conditions. Pairs like *aspire-aspirin* or *lizard-wizard* did not prime at any SOA. These results, though not unequivocal, suggest that morphological effects can be detected in visual priming tasks at short SOAs that cannot simply be attributed to either semantic or orthographic factors. This is also consistent with results recently reported for French by Longtin, Segui, and Hallé (2003) and for Arabic by Boudelaa and Marslen-Wilson (2005). It is less straightforward, however, to draw the conclusion that this therefore reflects the role of an independent level of morphemic representation in early visual word-recognition processes.

In particular, a recent study by Gonnerman and Plaut (2000) calls into question the claim that early effects of morphological structure can straightforwardly be interpreted as evidence for independent morphological processes. Approaching these issues from a connectionist perspective, where morphological effects are seen as a property of the interaction between semantic and form-based factors (e.g., Seidenberg & Gonnerman, 2000), Gonnerman and Plaut (2000) predicted that priming effects between derivationally related word-pairs would primarily reflect the degree of semantic overlap between these pairs. Using a short SOA of 36 ms, they found stronger priming for highly semantically related pairs, such as *boldly-bold*, than for moderately semantically related pairs like *lately-late*, where both pairs are viewed as morphologically related. There was no priming for pairs like *hardly-hard*, which were classed as morphologically and ortho-graphically related but semantically unrelated. These results were taken to support the claim that morphological priming effects primarily derive from meaning overlap between derived words.

These two experiments, using a similar methodology, produce very different results, with Rastle et al. (2000) finding early effects of morphology which cannot simply be attributed to the influence of form and meaning overlap, while the Gonnerman and Plaut study (2000) found that the effects of morphology were attributable to semantic similarity. We carried out the present set of studies in an attempt to reconcile these different results, since the two experiments are sufficiently different in important ways as to make it difficult to compare them directly. The Rastle et al. (2000) study manipulated time-course as a way of exploring different levels of lexical representations, but did not manipulate the critical variable of degree of semantic relatedness. The Gonnerman and Plaut (2000) study focused on graded priming effects as a function of degree of semantic similarity, but did not take the temporal dynamics of the word recognition process into account. In the first set of studies reported here we manipulated both SOA and degree of semantic relatedness, presenting the same set of stimuli at three prime exposure durations. In Experiments 1a and 1b we examined the two shorter SOAs from these earlier studies the 36 ms SOA used by Gonnerman and Plaut (2000) and the 48 ms SOA used by Rastle et al. (2000). To complete the set of comparisons we extended this in Experiment 1c to the 72 ms SOA also used by Rastle et al. (2000). At each SOA we tested claims for graded priming effects as a function of semantic overlap, varying the degree of semantic similarity for sets of morphologically decomposable prime-target pairs (i.e., potentially sharing a stem) and for pairs that were not morphologically decomposable.

This led to a design that manipulated orthographic, morphological, and semantic links between prime-target pairs across six experimental conditions. Note that it is necessary to distinguish here between potential and actual morphological linkage. Pairs like *scandal-scan*, although they apparently share the stem *scan*, are neither actually nor potentially morphologically related, since the syllable *dal* is not a derivational affix in English, so that there is no linguistic basis for segmenting *scandal* into a stem + affix structure. Intermediate pairs such as *archer-arch* are potentially morphologically related, since *archer* can be segmented into the stem *arch* and the productive derivational affix *-er*, and this pseudo stem has the same surface form as the free stem *arch*, occurring as the target. However, these words could not be actually morphologically related, in the sense of sharing the same underlying morpheme, since this would give the wrong meaning for *archer*. Semantically transparent pairs, like *bravely-brave*, however, are both potentially and actually morphologically related, since the morpheme brave is arguably shared across derived and stem forms (and such pairs prime robustly in overt priming tasks, as opposed to pairs like *archer-arch*).

The resulting six conditions (see Table 1) were as follows: (1) Related only in form and not morphologically decomposable (*scandal-scan*: – M – S + O). (2) Related in form, morphologically decomposable and potentially morphologically related, but not semantically related (*archer-arch*: + M – S + O). (3) Related in form, morphologically decomposable and potentially morphologically related, and semantically related at an intermediate level of relatedness (*barely-bare*: + M midS + O). (4) Only semantically related at an intermediate level of relatedness (*attach-glue*: – M midS – (O). (5) Related in form, morphologically decomposable and morphologically related, and highly related in meaning (*bravely-brave*: + M + S + O). (6) Only highly semantically related (*accuse-blame*: – M + S –O). Note that amount of priming is evaluated throughout by presenting the same targets preceded by an unrelated (–M –S –O) control prime.

**Table 1:** Experiment 1a-c: Test conditions and sample stimuli

Condition	Example prime-target pair	Morphological decomposability	Semantic relatedness	Orthographic overlap
1	scandal-scan	−M	−S	+O
2	archer-arch	+M	−S	+O
3	barely-bare	+M	MidS	+O
4	attach-glue	−M	MidS	+O
5	bravely-brave	+M	+ S	+O
6	accuse-blame	−M	+ S	– O

+/−M: Morphologically decomposable/not decomposable; +/−O: Orthographic overlap high/low; +/Mid/–S: Semantically highly related/moderately related/unrelated.

This semi-factorial set of contrasts allows us to ask three main questions: (a) Does degree of semantic relatedness affect priming and does this interact with apparent morphological effects? (b) Can priming for morphologically linked word-pairs be distinguished from priming attributable to form overlap or meaning overlap alone? (c) Do the effects of morphological, semantic, and orthographic factors differ across SOAs, consistent with recent evidence for an early stage of morphological-driven segmentation of complex forms?

In Experiment 2, using the same stimuli but in stem-derived order, we examine further questions about the nature and locus of morphological masked priming effects, raised by this first set of experiments.

## EXPERIMENT 1a-c

### Method

#### Materials and design

There were 24 prime-target pairs in each of six experimental conditions (see Table 1). All targets were monosyllabic free morphemes. In four conditions the prime and target shared orthography (Conditions 1, 2, 3, 5), with targets embedded in their primes at word onset, and the amount of form overlap (defined as the proportion of letters in a target relative to the number of letters in the prime) matched across the conditions.

The morphological status of the primes was determined using the CELEX English lexical database (Baayen, Piepenbrock, & Gulikers, 1995) and a modified version of the criteria set out in Marslen-Wilson, Tyler, Waksler, and Older (1994). Word-pairs were classified as morphologically decomposable (+ M), and therefore potentially morphologically linked, if the derived form had a recognisable affix (as listed by Marchand, 1969), which was attached to a potential stem (a form which could stand on its own as a real word). This was the case for the primes in conditions 2, 3, and 5. In Condition 1, the form did not terminate in a potential affix (e.g., ‘-dal’ in *scandal* is not an English suffix). However, all Condition 1 pairs had a similar orthographic and phonological structure to the (+ M) forms, with the prime being made up of a potential stem followed by a phonologically separable second syllable, and with the pseudo-stem in the target having the same pronunciation as in the prime.

The manipulation of semantic relatedness was based on pre-tests, where 14 participants rated a large set of word-pairs on a 9-point scale, with 1 being ‘not related at all in meaning’ and 9 being ‘very related in meaning’. Semantically unrelated pairs (+S Conditions 1 and 2) were rated between 1 and 3, medium related pairs (MidS Conditions 3 and 4) between 3 and 6, and highly semantically related pairs (– S Conditions 5 and 6) between 6 and 9. The semantically related pairs in Conditions 4 and 6 were neither orthographically nor morphologically related. The test words are listed in Appendix A.

A set of 144 unrelated control prime-target pairs were created by pseudo-randomising the primes around the targets. Experimental items were mixed with 72 pairs of unrelated filler words and 216 word nonword pairs (12 pairs in which nonwords were embedded in real words whose endings were not affixes in English (e.g., *tragedy-trag*), similar to the real words in condition 1; 36 pairs with nonwords embedded in real words whose ending is an affix in English (e.g., *derby-derb*), matching the morphologically related items; and 168 pairs where the nonword target was primed by an orthographically unrelated word (e.g., *garlic-teg*), as in the semantically related conditions and filler items). Nonword targets were orthographically and phonologically legal sequences. This generated a total of 432 prime-target pairs, with an equal number of word and non-word targets. Test targets were divided into two lists of 72 items. For each SOA (Experiments 1a-c) half of the subjects saw the first list in which targets were preceded by related primes and the second list in which targets were preceded by unrelated words while the other half saw the reverse order. Filler items and non-words were the same in both lists. All the targets appeared once in each list and had the same order in both. Subjects were given 30 practice trials at the beginning of the experiment.

Primes and targets were matched as far as possible across conditions for number of letters, lemma and word form frequency (CELEX database, Baayen et al., 1995) and neighbourhood (N) size. Average values for these variables across conditions are shown in Table 2. Because of the constraints imposed by the main design variables we could not fully match length across the conditions, *F*(5, 138)=4.73, *p*<.01 for primes, and *F*(5, 138)=5.3, *p*<.01 for targets. Primes were slightly shorter in Conditions 4 and 6, and targets were shorter in Condition 1 (– M – S + O). There was also some variation in neighbourhood size (N) for primes *F*(5, 138)=2,87, *p*<.05, although N was generally very low here (averaging 2.1). In addition, semantic relatedness could not be perfectly matched across conditions within a category (low, intermediate, high relatedness). Condition 1 (– M – S + O) had a lower relatedness value than Condition 2 (+ M – S + O), *t*(46)= −5.6; *p*<.01, and the ratings for Condition 4 (– M midS – O) were lower than those for Condition 3 (+ M midS + O), *t*(46)= −2.23, *p*<.05.^1^

**Table 2:** Experiment 1a-c: Stimulus properties across test conditions

			Length		Lemma frequency		N size		Word-form frequency	
						
Condition	Sem-rel	% form overlap	prime	target	prime	target	prime	target	prime	target
1. −M −S+O scandal-scan	1.5	.57	6.4	3.7	13.1	16.5	1.1	9.9	9.6	11.0
2. +M −S+O archer arch	2.2	.66	6.3	4.1	8.0	16.7	2.4	9.7	5.8	10.5
3. +M midS +O barely-bare	4.6	.69	6.3	4.3	7.4	22.0	2.1	7.5	6.8	11.9
4. −M midS +O attach-glue	5.1	n/a	5.9	84.4	12.4	24.5	3.0	6.3	5.5	10.5
5. +M +S +O bravely-brave	7.7	.69	6.8	4.6	7.4	21.8	0.9	6.7	6.6	15.0
6. −M +S −O accuse-blame	7.8	n/a	5.5	4.3	14.2	23.9	3.1	8.6	7.9	14.2

+/−M: Morphologically decomposable/not decomposable; +/−O: Orthographic overlap high/low; +/Mid/−S: Semantically highly related/moderately related/unrelated; Sem-rel= semantic relatedness.

#### Procedure

Subjects were told that they would see a series of letter strings and should decide as quickly and accurately as possible whether each string was a real word in English or not. They pressed one response key if the sequence was a word and another if it was a non-word. They were told that hash marks preceded each string but not of the existence of primes. A forward mask (hash marks) was displayed for 500 ms followed by a prime, then a target presented for 200 ms. Prime exposure duration (SOA) was 36 ms for Experiment 1a, 48 ms for Experiment 1b, and 72 ms for Experiment 1c, with different subjects tested at each SOA. Targets were in upper case and primes in lower case. Stimulus presentation and data recording were controlled by DMDX software (Forster & Forster, 2003) running on Pentium II PCs.

#### Subjects

All subjects were recruited from the Centre for Speech and Language’s subject pool; 33 subjects were run at the 36 ms SOA (Experiment 1a), a further 33 at the 48 ms SOA (Experiment 1b), and 30 at the 72 ms SOA (Experiment 1c), for a total of 96. All were native speakers of British English and were paid £5 for their participation.

### Results

To allow for a more compact presentation of the results, and since SOA turned out not to be a major determinant of the effects, we present Experiments 1a-c in a combined analysis, rather than treating them separately in sequence.

#### (a) Reaction time analyses

**Figure 1:**
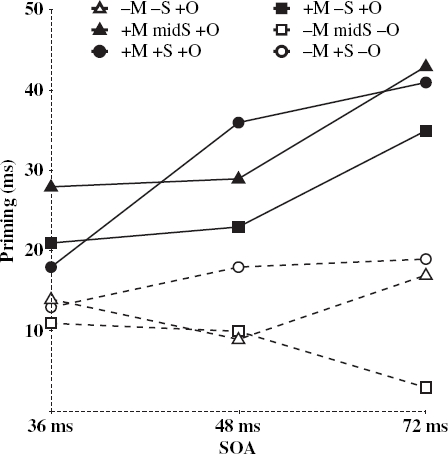
Mean priming effects at each SOA for Conditions 1–6.

All errors (7.9%) and time-outs (0.2%), defined as responses longer than 2000 ms, were removed. Two items, *curt* and *pious*, were excluded because of error rates over 50%. The data were then analysed in two ways, using conventional ANOVA techniques and using multi-level regression (e.g., Baayen, Tweedie, & Schreuder, 2002). We report first the conventional analyses, conducted on subject and items means. The raw RTs for all correct responses were inversely transformed (Ratcliff, 1993) and entered into a mixed-design analysis of variance with four factors: priming (primed and unprimed), version (two levels), condition (six levels) and prime duration (SOA; 36, 48, and 72 ms). In the subjects analysis (*F*_1_), condition and priming were treated as repeated measures and version and SOA as independent factors. In the items analysis (*F*_2_), SOA and priming were treated as repeated factors and version and condition as independent factors. Mean RTs and error rates are summarised in Table 3. Priming effects (expressed as unprimed minus primed RT) by condition and SOA are shown in Figure 1.

**Table 3:** Experiment 1a-c: Harmonic mean RTs (ms) and error rates (%)

		36 ms		48 ms		72 ms	
				
Condition	SOA Prime Type	RT (error)	Priming (ms)	RT (error)	Priming (ms)	RT (error)	Priming (ms)
1. −M −S +O (scandal-scan)	Test	525 (12%)	14	539 (12%)	9	563 (15%)	17
	Control	539 (10%)		547 (12%)		580 (10%)	
2. −M −S+O (archer-arch)	Test	507 (7%)	21 **	513 (5%)	23 **	523 (6%)	35 **
	Control	528 (6%)		536 (7%)		558 (7%)	
3. −M midS+O (barely-bare)	Test	504 (9%)	28 **	505 (7%)	29 **	525 (5%)	43 **
	Control	533 (12%)		534 (11%)		567 (12%)	
4. −M midS−O (attach-glue)	Test	527 (11%)	11	532 (8%)	10	564 (8%)	3
	Control	538 (11%)		542 (9%)		567 (10%)	
5. +M+S+O (bravely-brave)	Test	495 (5%)	18*	493 (3%)	36**	505 (2%)	41**
	Control	513 (5%)		529 (6%)		547 (4%)	
6. −M+S−O (accuse blame)	Test	517 (6%)	13 (*)	523 (6%)	18*	537 (3%)	19 *
	Control	530 (6%)		541 (7%)		556 (9%)	

+/−M: Morphologically decomposable/not decomposable; +/−O: Orthographic overlap high/low; +/Mid/−S: Semantically highly related/moderately related/unrelated.

There was a strong main effect of priming with faster RTs to primed (522 ms) than unprimed (544 ms) targets, *F*_1_(1, 90)=147.60, *p*<.01; *F*_2_(1, 130)=116.00, *p*<.01. There was a main effect of SOA by items only *F*_1_(2, 90)=1.43, *p*>.1; *F*_2_(2, 260)=100.86, *p*<.01 with slower RTs at longer SOAs, and no interaction with priming: SOA by prime, *F*_1_(2, 90)=1.15, *p*>.1, *F*_2_(2, 260)=2.05, *p*>.1. There was a main effect of condition *F*_1_(5, 450)=41.69, *p*<.01, *F*_2_(5, 130)=3.51, *p*<.01, and a significant condition by priming interaction, indicating that priming effects varied across conditions *F*_1_(5, 450)=7.50, *p*<.01; *F*_2_(5, 130)=6.05, *p*<0.1. In further analyses, we investigated the effects of the three principal factors of morphological, semantic, and orthographic relatedness.

These analyses show that the dominant factor is morphological decomposability, with very similar effects across SOAs. The strongest priming effects at each SOA were for the three (+ M) conditions (see Figure 1). Collapsing across SOAs, the conditions which showed the most robust effects were those where prime and target were potentially morphologically related. This held irrespective of degree of semantic relatedness, as shown in a series of planned comparisons. These revealed significant priming effects for Condition 2 (*archer-arch*) + M – S + O, with *F*_1_(1, 90)=34.29, p <.01; *F*_2_(1, 22)=28.17, *p*<.01, Condition 3 (*barely-bare*) + M midS + O, with *F*_1_(1, 90)=67.78, *p*<.01; *F*_2_(1, 22)=48.22, *p*<.01, and for Condition 5 (*bravely-brave*) + M + S + O, with *F*_1_(1, 90)=83.91, *p*<.01; *F*_2_(1, 22)=47.45, *p*<.01. An analysis of these three conditions together showed a significant main effect of priming, *F*_1_(1, 90)=131.25, *p*<.01; *F*_2_(1, 66)=121.87, *p*<.01, but no interaction of prime with condition, *F*_1_(2, 180)=1.30, *p*>.1; *F*_2_<1. Degree of semantic overlap between prime and target did not modulate the size of the priming effect for (+ M) items. This also held for each SOA individually (all *p*>.1 for the relevant prime by condition interaction). Unlike the pattern reported by Rastle et al. (2000), there was no sign of a drop off in priming for the + M – S + O (*archer-arch*) condition at the 72 ms SOA. In general, (+ M) priming effects showed a tendency to increase over SOAs, with a marginal interaction between prime and SOA, *F*_1_(2, 90)=2.54, *p*=.08; *F*_2_(2, 132)=3.84, *p*<.05.

More generally, comparing the three morphologically related (+ M) conditions with the three (– M) conditions (1, 4, 6), we see larger priming effects (30 ms) for the (+ M) conditions compared with the (– M) conditions (13 ms), reflected in a significant interaction between priming and morphology, *F*_1_ (1,90)=28.44, *p*<.01; *F*_2_(1,138)=25.77, *p*<.01). This also holds when the three (+ M) conditions are compared just to the form-based control (Condition 1), with *F*_1_(1, 90)=12.14, *p*<.01; *F*_2_(1, 91)=14.18, *p*<.01, or to the two semantic conditions - for Condition 4, *F*_1_(1, 90)=26.46, *p*<.01; *F*_2_(1, 92)=19.24, *p*<.01; for Condition 6, *F*_1_(1, 90)=11.01, *p*<.01; *F*_2_(1, 91)=7.23, *p*<.01.

Form overlap alone (Condition 1) did not show significant priming effects at any SOA. Collapsing over SOAs, the overall effect of 13 ms was only marginally significant, *F*_1_(1, 90)=12.43, *p* <01; *F*_2_(1, 21)=3.59, *p*=.07. Semantic relatedness alone produced mixed effects. The midS set (Condition 4) showed no priming at any SOA, nor any overall effect, collapsing across SOA, with *F*_1_(1, 90)=6.29, *p* <05; *F*_2_(1, 22)=2.41, *p* >.1. The highly semantically related set (Condition 6), in contrast, did show a significant overall effect of 17 ms, *F*_1_(1, 90)=20.42, *p*<01; *F*_2_(1, 21)=12.55, *p*<.01), with marginal priming at SOA 36, *F*_1_(1, 31)05.26, *p*<05; *F*_2_(1, 21)=4.09, *p*=.06, and stronger effects at SOA 48, *F*_1_(1, 31)=8.56, *p* <01; *F*_2_(1, 21)=4.91, *p*<.05, and SOA 72, *F*_1_(1, 28)=7.02, *p*<05; *F*_2_(1, 21)=5.98, *p*<.05.

In a second set of analyses, we adopted a regression-based approach, running multi-level regression analyses on individual responses (Baayen et al., 2002). This allows us both to mitigate the potential biases introduced by a factorial design (e.g., Ford, Marslen-Wilson, & Davis, 2003), and to avoid the loss of statistical power due to the use of item and subject means (e.g., Baayen, 2004, 2007). We performed a multi-level linear regression with log response latency as the dependent variable and log prime and target frequency and factors of morphological, semantic, and orthographic relatedness as predictors. Consistent with the results of the classical ANOVA, these analyses showed no interaction of priming with SOA, either in the twoway interaction with priming, *F*(2, 12398)=1.23, *p*>.1, or in the three way interaction with priming and condition, *F*(10, 12398)=0.50, *p*>.1. Since no effect of SOA was found, all subsequent analyses were performed without this variable in the model. These analyses revealed a significant effect of morphological relatedness on priming, *F*(2, 12418)=62.47, *p*<.0001, but no interaction of this effect either with semantic relatedness, *F*(4, 12418)=0.35, *p*>.1, or with orthographic overlap, *F*(4, 12418)=1.25, *p*>.1. This was after partialling out the effects of prime and target frequency, *F*(1, 12418)=14.34, *p*<.001 and *F*(1, 12418)=402.24, *p*<.0001, respectively).^2^ The same pattern of results emerged in analyses that included both item and subject variance as cross-random effects, further confirming the robustness of the current findings and suggesting that the absence of an interaction between semantic relatedness and morphological priming was not due to a lack of statistical power.

#### (b) Error analyses

The error data were only analysed using the classical ANOVA approach. This analysis showed main effects of priming, *F*_1_(1, 90)=7.98, *p*<.01; *F*_2_(1, 130)=9.71, *p*<.01, and condition, *F*_1_(5, 450)=29.55, *p*<.01; *F*_2_(5, 130)=2.96, *p*<.05, but not of SOA. A marginally significant priming by condition interaction, *F*_1_(5, 450)=3.53, *p*<.01; *F*_2_(5, 130)=2.04, *p*=.08, reflects the fact that the error rate was higher for unprimed than primed items (8.1% vs. 6.1%) except in Condition 1 (– M –S + O). There were no further interactions.

### Discussion

Many studies have shown that prior exposure of a morphologically related word facilitates the recognition of a subsequently presented item (Feldman, 2000; Frost, Forster, & Deutsch, 1997; Marslen-Wilson et al., 1994). However, words in English that share morphological structure usually also share form and meaning, and it is difficult to assess the extent to which this facilitation is specifically due to morphological factors. In the first three experiments we used masked primes presented at different SOAs to examine the effects produced by these different dimensions of relatedness, asking whether morphological priming can be observed separately from the effects produced by shared form and shared meaning in early word recognition. The pattern of results not only provides answers to the three questions asked in the introduction, but also has important implications for how one should interpret the presence of morphological effects in masked priming.

The first question followed up the claim by Gonnerman and Plaut (2000) that the degree of semantic relatedness between words determines the amount of priming, and that morphological effects primarily arise from meaning overlap between derived words. The results here are unequivocally inconsistent with this claim. First, we see significant priming between morphologically decomposable pairs even when these have no semantic relationship, as in Condition 2. Second, despite the fact that reliable semantic effects were found in Condition 6 for highly related + S–M pairs like *accuse-blame*, there was no interaction between semantic relatedness and amount of priming in the three + M conditions (2, 3, and 5), where form overlap is held constant. Most tellingly, priming in the MidS + M condition, for pairs like *barely-bare*, averaged a robust 33 ms, which differs significantly from the 8 ms priming effect in the matched MidS – M condition, for pairs like *attach-glue*, *F*_1_(1, 90)=19.77, *p*<.01; *F*_2_(1, 44)=16.48, *p*<.01. This shows that even when semantic factors alone are unable to generate any priming, morphological factors can generate priming effects that are as strong as those in any of the other + M conditions. Even if morphological effects are argued to reflect the joint contribution of form- and meaning-based constraints, rather than either one of these in isolation, we still expect to see graded effects of semantic relatedness on priming between + M pairs that are matched for amount of form overlap.

These results also address the second question, asking whether priming for morphologically linked word-pairs can be distinguished from priming attributable to form or meaning overlap alone. Morphologically based priming proves to be clearly dissociable from semantically based priming. Where form overlap is concerned, we see no significant effects at any SOA, and significantly stronger priming for the three (+ M) conditions compared with the pure form Condition 1. Similar conclusions emerge from the regression analyses, which could detect no relationship between amount of priming and either semantic relatedness or amount of orthographic overlap. At each SOA, it is morphological decomposability that is the significant predictor of priming.

The third question concerned the time-course of morphological, semantic, and orthographic contributions to the word recognition process, and the possible evidence for an early stage of morphologically-driven segmentation. Morphological effects are robustly significant at the earliest SOA, though they show a tendency to increase over SOAs (from 22 ms at SOA 36 to 40 ms at SOA 72). These effects, consistent with an early segmentation stage, are not modulated by semantic relatedness (or, therefore, by the status of the primes as genuinely morphologically structured). Unlike Rastle et al. (2000), we do not see a drop off in the effects for the + M – S(*archer-arch*) condition at the 72 ms SOA. This may reflect between-experiment variability in the degree of masking at this SOA (see also Tzur & Frost, 2007), since to the extent that participants become aware of the prime, we expect priming for these pairs to disappear (cf. Longtin et al., 2003; Feldman & Soltano, 1999). Pure semantic relatedness shows weak effects at the shortest SOA, increasing in statistical robustness at longer SOAs, but remaining significantly weaker than the morphological effects. Purely form-based effects are weak throughout, and show no evidence of changing over SOAs. We conclude from this that morphological effects are strongly present from our earliest measurement point, consistent with Rastle et al. (2000) for English and with recent results for Arabic (Boudelaa & Marslen-Wilson, 2005), but there is no clear evidence here that other effects, if present, have markedly different time-courses.

A noteworthy aspect of the current results, although consistent with Gonnerman and Plaut's (2000) original report, is that we do see significant semantic priming effects at shorter SOAs – in particular the 18 ms effect at SOA 48. Priming effects between pairs that are only semantically related are uncommon in the masked priming literature, although some cases have been reported (e.g., Bodner & Masson, 2003; Perea & Gotor, 1997; Perea & Rosa, 2002). However, the relevant point for the current research is not whether or not we observe semantic priming, but whether semantic factors determine masked morphological priming. The answer for this study is that clearly they do not.

The further important implication of the results is that they bring into question exactly what masked priming is telling us about the structure of lexical representations, given the potential locus of the observed effects at an early segmentation stage. This is the issue we turn to below.

#### Morphological masked priming and levels of lexical representation

Conventionally, morphological priming in languages like English is thought to reflect repeated access to a morpheme shared by prime and target (e.g., Marslen-Wilson et al., 1994). Under conditions where primes are fully visible or audible – such as cross-modal repetition priming – priming is obtained between pairs such as *agreement-agree*, which are transparently morphologically related, but not between pairs like *apartment-apart*, which share a similar form relationship but are not synchronically morphologically related. This is taken to reflect different relationships between lexical representations, where *agreement* and *agree* share the common morpheme {*agree*}, but where *apartment* and *apart* do not share the morpheme {*apart*}. This account does not require the segmentation of a complex prime into its morphological components as part of the access process. Morphological effects reflect the structure of the underlying lexical representation, not how it is accessed.

In earlier masked priming studies investigating morphological relationships, a similar logic has applied. If *calmness* primed *calm* or *hunter* primed *hunt*, this was because of shared morphemes in prime and target, bringing savings in response times to the target. This interpretation, however, is undermined by several recent masked priming results, including those we report here (e.g., Dominguez, Segui, & Cuetos, 2002; Forster & Azuma, 2000; Longtin et al., 2003; Rastle et al., 2000; Rastle, Davis, & New, 2004). These show, across several languages, that strong priming (masked but not overt) can be obtained between pairs like *archer-arch and treaty-treat* where there is no underlying morphological link between prime and target. If *archer* primes *arch*, this cannot be because the lexical representation of *archer* contains the same morpheme {*arch*} that is then re-activated by the target *arch*.

These studies point instead to an early obligatory segmentation of all complex derived words that is blind to their semantic transparency or opacity (cf. Taft & Forster, 1975). When *archer* is encountered, this is segmented into the potential stem + affix pair {*arch*} + {-*er*}, and stored form-based representations for these elements are activated. This leads to priming when *arch* is presented as target. Our results suggest that this may be the primary mechanism at work in masked priming, since the pattern of effects for morphologically unrelated *archer-arch* pairs did not differ from those for related pairs like *bravely-brave* at all SOAs tested. The critical factor seems to be the decomposability of a complex prime – whether it can be pre-lexically segmented into a stem and an affix, such that the stem matches in form to the subsequently presented target word.

This raises the further question of whether these are morphological effects at all, or whether any complex prime containing the target will prime successfully. Recent results for French suggest that morphological factors can be critical. Using an SOA of 43 ms, Longtin *and* Meunier (2005) show that non-word primes (such as *rapidifier*) can prime their pseudo-stem (*rapide*, ‘rapid’) just as well as transparent real-word primes (*rapidement*, ‘rapidly’), but only if the pseudo-stem co-occurs with an existing French suffix. Thus *rapiduit*, where -*uit* is not a possible suffix in French, does not prime *rapide*. These results not only support the early segmentation account of morphological priming (pseudo-words, by definition, cannot have a stored lexical representation), but also show that this early segmentation is sensitive to morphological factors. Only if the potential stem is paired with an actual suffix in the language do we see priming.

The story is less clear for English, where it is harder to get straightforward differences between prime-target pairs decomposable on morphological grounds and those where the relationship is purely orthographic (e.g., Forster & Azuma, 2000; Rastle & Davis, 2003; Rastle et al., 2004). Forster and Azuma (2000) only achieve this at a long SOA, with word-like nonwords, and only for a subset of the items. A concern about the Rastle and Davis (2003) and Rastle et al. (2004) results is the role of phonological overlap, with the stimulus pairs in these experiments being constructed on the basis of orthographic criteria alone, thus allowing an uneven distribution of phonologically disparate pairs like *rabbit-rabbi, united-unit, and plumage-plum*.^3^ Taft *and* Kougious (2004) do find priming between bisyllabic pairs sharing a bound stem (as in *virus-viral*) independent of phonological overlap, but since they did not control for the affixal status of the second syllable in their materials, this result is hard to interpret in the present context. In the experiment here, where primes and targets match phonologically as well as orthographically, we see a less clear-cut (though still significant) difference between form-based controls and the (+ M) conditions. It is likely that further research is needed to pin down exactly which factors control early segmentation in English visual word recognition.

Nonetheless, an account along these lines for English would be entirely consistent with the demonstration that morphological effects in masked priming operate independently of semantic factors. If it is early segmentation that drives these effects, then the actual lexical representations of complex prime words – in terms of both their morphological and semantic properties – will be irrelevant to whether or not priming is obtained. We can test this claim in a further experiment, by reversing the order in which morphologically decomposable prime/target pairs are presented in a masked priming task.

## Experiment 2

In Experiments 1a-c, the complex derived or pseudo-derived form was presented first, as in pairs like *archer-arch, hardly-hard, and bravely-brave*. These [+ M] complex forms are argued to be decomposed into potential stem + affix sequences, leading to the facilitation of lexical decision responses to the subsequently presented stem or pseudo-stem (*arch, hard*, or *brave*). But because these stem targets are monomorphemic, this leaves ambiguous the locus of this facilitation. If a form like *arch* is represented at a peripheral, early stage of the system, where blind decomposition is thought to take place, then the activation of this representation when *archer* is presented (and decomposed) could lead to priming when *arch* is immediately presented as target. However – or in addition – priming could also be mediated at a central level of lexical representation, where *arch* (and its full lexical properties) must also be represented. The transient segmentation of *archer* into *arch* + *-er*, under masked priming conditions, could lead to the activation of arch at a central lexical level, and hence generate priming effects. Similar ambiguities hold for pairs like *hardly-hard and bravely-brave*.

This ambiguity can be tackled by reversing the order of prime and target, so that the stem (or pseudo-stem) is the prime and the complex form is the target. If priming is mediated through activation of central lexical representations, then there should be clear effects of the properties of these representations, with less priming for prime-target pairs like *arch-archer* than for transparent pairs like *brave-bravely*. In the latter case, the central representation for *bravely* arguably incorporates the morpheme *brave*, so that the prior occurrence of *brave* should strongly facilitate responses to *bravely* – as shown in earlier cross-modal and auditory-auditory priming experiments using the stem/derived order with an overt rather than masked prime (Marslen-Wilson et al., 1994; Marslen-Wilson & Zhou, 1999). In these tasks, the outcome is assumed to reflect the morphological and semantic properties of central lexical representations, since pairs like *hardly-hard* do not show priming, reflecting their unrelatedness at this level of the system.

On this analysis, *arch* could not be an effective prime of archer, because the lexical representation of *archer* is as a morphologically simple word, which is not linked to the morpheme *arch*. As far as we are aware, there is no research using overt priming tasks with pseudo-suffixed stem-derived pairs like *arch-archer*. The evidence from pseudo-prefixed stem-derived pairs like *strain-restrain*, however, is that this leads to significant interference effects in cross-modal priming (Marslen-Wilson et al., 1994). This is consistent with the view that pairs like *strain* and *restrain* are separate lexical items, where prior presentation of *strain* may lead to the attempt to interpret *restrain* as the non-existent *re-strain*, generating interference effects in lexical decision. Similar effects could play a role in pseudo-suffixed pairs as well. Note that transparent prefixed stem-derived pairs like *sincere-insincere* behave like transparent suffixed pairs, and show robust priming in cross-modal tasks (e.g., Marslen-Wilson et al., 1994).

In summary, if morphological masked priming is driven by repeated activation of shared morphemes at a central lexical level, then we should see clear differences in the amount of priming as a function of the transparency of stem-derived pairs - priming should only be obtained for the (+ M + S) pairs like *brave-bravely*. In contrast, if masked priming effects for morphologically decomposable pairs are primarily driven by activation effects at a peripheral, form-based level of representation, as argued above, then the differential effects should be much weaker, and we should see similar effects to those observed in Experiments 1a-c for the derived/stem presentation order.^4^ There should still be no priming for the Condition 1 pairs like *scan-scandal, since scandal* is not decomposable into the potential stem + affix pair *scan* + *-dal*, and therefore provides no point of contact with the representations activated by the prime *scan*. The three + M conditions (2, 3, 5) should all show priming, since the targets are all decomposable into stem + affix pairs, providing a basis for facilitation based on repeated activation of the same morpho-orthographic component by prime and target. If the properties of central lexical representations also contribute to these effects, then priming should be strongest for the [+ S] pairs like *brave-bravely* in Condition 5, and weakest for the [– (S] pairs in Condition 2.

To allow the cleanest comparison with the derived-stem tests in Experiments 1a-c, we ran the same set of materials in Experiment 2, including the two semantics-only conditions (– M midS, – M + S), so that the overall stimulus environment remained constant. Given the absence of strong SOA effects, we ran the study at just one SOA, choosing the 48 ms SOA as a prime duration that is thought to deliver unambiguously masked priming.

### Method

#### Materials and design

The experiment used the same word stimuli as in Experiments 1a-c, with only the order of presentation reversed (e.g. *brave-bravely* instead of *bravely-brave*), and with the same six experimental conditions with 24 prime-target pairs each (see Table 1). All the targets were monosyllabic free morphemes. Primes and targets were matched across conditions on length, word and bigram frequency, and N size as closely as possible (see Table 2).^5^ One hundred and forty-four unrelated control prime-target pairs were created by pseudorandomising the primes around the targets. Experimental items were mixed with 72 pairs of unrelated filler words and 216 new nonword pairs matching the structure of test items (nonword targets and test targets were also matched for bigram frequency). The nonword pairs consisted of 12 pairs in which nonword primes were embedded in nonword targets whose endings were not affixes in English (e.g., *donk-donkel*), similar to the real words in condition 1; 48 pairs of nonword pairs where nonword stems were embedded in nonwords whose ending is an affix in English (e.g., *chont-chontly*) to match the morphologically related test items; and 168 pairs where the nonword target was primed by an orthographically unrelated word (e.g., *delay-swom*), paralleling the semantically related conditions and the filler items. This generated a total of 432 prime-target pairs, with an equal number of word and non-word targets. Test pairs were rotated across two versions such that they were preceded by a related prime in one version and an unrelated prime in the other. Filler items and non-words were the same in both lists. All the targets appeared once in each list and had the same order in both. Subjects were given 30 practice trials at the beginning of the experiment.

#### Procedure

We used the same procedure as in the first three experiments, but with no variation in SOA. Primes were presented for 48 ms, preceded by a forward mask of hash marks displayed for 500 ms. Targets were presented for 200 ms. Subjects were asked to make a lexical decision to each target.

#### Subjects

Thirty-six subjects from the Centre for Speech and Language subject pool participated in this study (19 subjects on version 1 and 17 subjects on version 2). All were native speakers of British English, with normal or corrected-to-normal vision. Subjects were paid £5 for their participation.

### Results and discussion

All errors (7.6%) and timeouts (0.1%), defined as RTs>2000 ms, were removed. Three subjects had error rates above 20% and were discarded from the analysis. Three items (*dollop, beefy, frond*) elicited more than 50% errors and were also excluded. Raw RTs were inverse transformed and entered into a mixed-design ANOVA, as in Experiments 1a-c, but without the factor of SOA. Overall condition means and error rates are given in Table 4.

The results showed a main effect of priming, *F*_1_(1, 34)=34.5, *p*<.01; *F*_2_(1, 93)=27.3, *p*<.01, with lexical decision for primed words 17 ms faster than for unprimed words (577 and 594 ms, respectively). The main effect of condition was significant by subjects but not by items *F*_1_(5, 170)=3.7, *p*<.0.1; *F*_2_<1, and there was no interaction between priming and condition (*F*_1_, *F*_2_<1).

Our main focus here is the results in the three (+ M) conditions. Analyses of each individually shows significant priming effects in Condition 2 (+ M – S + O; *arch-archer*), with *F*_1_(1, 34)=8.3, *p*<.01; *F*_2_(1, 16)=7.5, *p*<.05, and Condition 5 (+ M + S + O; *brave-bravely*), with *F*_1_(1, 34)=13.0, *p*<.01; *F*_2_(1, 16)=5.2, *p*<.05, and marginally significant priming in Condition 5 (+ M midS + O, *bare-barely*) with *F*_1_(1, 34)=3.2, *p*=.08; *F*_2_(1, 15)=6.7, *p*<.05. The amount of priming in each condition is very similar, ranging between 17 and 21 ms, and an analysis of variance for these three conditions on their own shows a main effect of priming *F*_1_(1, 34)=20.1, *p*<.01; *F*_2_(1, 68)=12.6, *p*<.01, but no interaction with condition (*F*_1_, *F*_2_<1). There is no sign here of any effect of semantic relatedness, and therefore no sign that priming is modulated by the status of the target words as genuinely morphologically structured or not. The importance, nonetheless, of morphological decomposability, is highlighted by the absence of priming in the form-only Condition 1 (– M – S + O; *scan/scandal*), with *F*_1_(1, 34)=1.6, *p*>.1; *F*_2_<1.

**Table 4:** Experiment 2: Harmonic mean RTs (ms) and error rates (%)

Condition		Test prime	Control prime	Priming (ms)
1.	−M−S+O (scan-scandal)	581 (6.5)	589 (3.9)	7
2.	+M−S+O (arch archer)	581 (6.0)	601(8.4)	20*
3.	+M mid S+O (bare barely)	572 (6.0)	589 (6.8)	17 (*)
4.	−M mid S−O (attach glue)	582 (7.0)	595 (6.7)	14 (*)
5.	+M+S−O (brave bravely)	567 (8.4)	588 (6.2)	21 *
6.	−M+S −O (accuse blame)	581 (6.5)	604 (6.9)	23 *

+/−M: Morphologically decomposable/not decomposable; +/−O: Orthographic overlap high/low; +/Mid/−S: Semantically highly related/moderately related/unrelated.

The two semantics-only conditions behaved in much the same way as before, with significant priming in the strong semantic overlap Condition 6 (– M + S – O; *blame-accuse*) with *F*_1_(1, 34)=10.9, *p*<.01; *F*_2_(1, 16)=6.9, *p*<.05. Priming in the intermediate semantic condition (–M midS – O; *glue-attach*) seemed slightly stronger here, *F*_1_(1, 34)=3.2, *p*=.08; *F*_2_(1, 15)=4.6, *p*<.05, but the numerical size of the effect, at 14 ms, was similar to the 10 ms effect at SOA 48 in Experiment 2 (see Table 3).

The error data were analysed in the same way as the latency data. Main effects of prime and condition were not significant (all Fs <1); and there was no significant prime by condition interaction *F*_1_(5, 170)=1.5, *p*>.1, *F*_2_<1.

Finally, we performed a multi-level regression on individual responses, along the same lines as for Experiments 1a-c. Log response latency was entered as dependent variable and log prime and target frequency and factors of morphological, semantic and orthographic relatedness as predictors. Again, the results replicate the findings from the classical ANOVA: there was a significant effect of morphological relatedness on priming, F(2, 4708)=4.06, *p*<.05, but priming was not modulated by the amount of semantic relatedness, F(4, 4708)=1.08, *p*>.1, nor by orthographic overlap, F(4, 4708)=0.76, *p*>.1. The same pattern held for analyses that included both items and subjects variance as cross-random effects.

The overall results are clear. There was significant priming for all of the (+ M) morphologically decomposable groups, confirming that this does not require the target word to actually contain the prime stem (or pseudo-stem) as part of its representation. Otherwise, *arch* would not have primed *archer*. Secondly, the amount of priming was not modulated by the semantic transparency of the relationship between a derived (or pseudo-derived) form and its stem (or pseudo-stem). Masked priming seems to be driven by decomposability, and not by the properties of central lexical representations.

## GENERAL DISCUSSION

This series of experiments helps to establish two important points about the structure of the visual word-recognition process, and how this is reflected in the masked priming task. The first is that visual word-recognition seems to incorporate, at an early stage in lexical access, the blind decomposition of potential morphologically complex forms into their component morphemic parts. The existence of a processing stage of this sort (originally proposed by Taft & Forster, 1975) has been reaffirmed in several recent papers, all using masked priming tasks with a range of opaque and pseudo-derived prime-target pairs, in languages that include English, French, and Spanish (e.g., Dominguez et al., 2002; Longtin et al., 2003; Rastle et al., 2000). The second point is that morphological priming effects in masked priming seem almost entirely driven by this early segmentation process, and do not interact either with the semantic properties of the prime and target or with the actual morphological relationship between them. In other words, morphological masked priming is not based on the repeated activation of shared morphemes between prime and target. The word *brave* does not prime *bravely* because both words share the morpheme {*brave*}, but for the same reason that *arch* primes *archer* i.e., because forms like *bravely* and *archer* can be segmented at an early stage of lexical access into two components, one of which matches the stem or pseudo-stem presented as the prime.

The critical evidence for these claims is the absence of any interaction with semantic relatedness in both derived-stem and stem-derived morphological priming. Morphological priming effects in masked derived-stem priming are not significantly modulated by variations in the semantic relatedness of prime and target – where semantic relatedness functions both as a measure of the semantic similarity between prime and target, and of the likelihood that prime and target share the same morpheme. In the set of derived-stem studies, the overall priming effect is numerically very similar for the three (+ M) conditions, averaging 26 ms for (+ M –S), 33 ms for (+ M midS), and 32 ms for (+ M + S). The statistical interaction between priming and semantic relatedness does not approach significance either overall or at any individual SOA. Experiment 2 reveals an equal lack of an interaction with semantics when we test in the stem-derived order. The absence of even a shred of statistical evidence for morphology/semantics interactions is confirmed by the results of the additional multi-level regression analyses for each experiment.

This set of findings tells us, first, that morphological priming effects in masked priming are not disguised semantic effects which can be explained in terms of the overlap in semantic properties of the prime word and the target word, generating priming in the same way that *accuse* might prime *blame*. Although we do see robust effects for pure (+ S) pairs like *accuse-blame*, these must rely on a different processing mechanism than masked morphological priming, since they seem to operate independently of priming generated by morphological decomposability. This decoupling of semantic relatedness from morphological priming refutes the persistent but weakly supported claim that morphological effects in priming tasks are simply byproducts of gradations in semantic relatedness between prime-target pairs (e.g., Gonnerman & Plaut, 2000).^6^ It is also inconsistent with the foundational assumptions of the underlying connectionist models assumed by these authors, where morphological effects are intrinsically semantic (as well as form-based) in nature.

The second important implication of the absence of semantic effects is what this tells us about the role of central lexical representations in masked morphological priming. This is because variation in semantic relatedness, for morphologically decomposable (+ M) pairs, not only addresses semantic issues, but also reflects the synchronic transparency of morphological relationships between derived forms and their potential stems. Transparent (+ S + M) pairs like *bravely-brave* or *calmness-calm* arguably share the same underlying morpheme, and extensive evidence from a variety of non-masked priming tasks shows robust priming for pairs like this (e.g., Marslen-Wilson et al., 1994). In comparison, semantically opaque (– S + M) pairs like *grateful-grate or rustic-rust* do not prime at all under these conditions. Not only does *rustic* not contain the morpheme {*rust*}, so that it will not directly activate the representation of rust, but also it is a cohort competitor (because it is a quite different lexical entry), and will generate competitive interference in recognition tasks (e.g., Gaskell & Marslen-Wilson, 2002). These robust differences between transparent and opaque derivational forms, which we see in priming tasks with overt visible or audible primes, are consistent with widely shared linguistic intuitions, which tell us that the relationship between *swim and swimmer or brave and bravely is quite different* from the relationship between *rustic* and *rust* or *grate* and *grateful*.

Since none of these differences modulate masked priming between (+ M) pairs, this strongly suggests that the task does not tap into levels of lexical representation where these kinds of distinctions are encoded. Even when the materials are presented in stem-derived order (as in Experiment 2), so that the complex form is fully visible to the participant, there is no effect of these higher-level lexical and semantic variables. If *rust* primes *rustic* as effectively as *brave* primes *bravely*, this cannot be because shared central morphemes are being sequentially activated in primes and targets.

The broader implication of these results is that multiple mechanisms are at work in the processing and representation of morphologically complex forms, and that different tasks tap into these mechanisms in selectively different ways. Masked priming seems to reflect, quite restrictively, the overlap between primes and targets generated by an early process of segmentation into stems and affixes. This is a lexically ‘blind’ process in the sense that it is not affected by the morphological and semantic properties of the derived and pseudo-derived full forms that are being segmented in this way. It is not, however, fully morphologically blind, because it is constrained by the status of the potential segments involved. If the form does not contain a potential derivational suffix, then priming is reduced or absent, as seen in the results here (for the clearest evidence to this effect, see Longtin & Meunier, 2005).

Priming effects where the prime is overt – whether in the visual or the auditory domain – are much more strongly influenced by the overlap between primes and targets in terms of their stored long-term lexical representations. Simple overlap between stems and pseudo-stems (as in cases like *rustic-rust* or *archer-arch*) does not generate priming. Instead, robust priming requires that the relationship between derivationally linked primes and targets should be semantically transparent, as in pairs like *darkness-dark* or *bravely-brave*. This is not because priming between these pairs is semantic in character, but because the central representations of prime and target share the same morpheme.

Both these morphologically characterised priming effects need to be distinguished from a third process underpinning semantic priming effects. These effects (between words that are semantically but not morphologically related, as in *accuse-blame*) are detectable both in masked and overt priming tasks, but in each case they can be distinguished from morphologically based effects. In the masked priming task – as demonstrated in the research reported here – semantic effects co-exist in parallel with morphological effects, but do not interact with them. Similarly, in overt priming tasks, semantic priming coexists with morphological priming under certain conditions, but can be clearly separated from it (for discussion see, e.g., Marslen-Wilson et al., 1994).

These considerations point to a system for lexical access and recognition where the morphemic properties of the input – containing stems, derivational morphemes, and inflectional morphemes are – of critical importance to this system from the earliest stages of the access process. In visual word recognition, where information about stems and affixes is made available to the system in parallel, the early segmentation process revealed in masked priming suggests that a first priority is to separate out potential stems and potential affixes. Emerging neuro-imaging results suggest that this process engages early stages of the visual processing stream, most likely associated with the visual word form area in inferior temporal cortex. Devlin, Jamison, Matthews, and Gonnerman (2004) reports masked priming effects in exactly this area, using an event-driven fMRI approach, for a mixture of semantically opaque and pseudo-derived stimuli (such as *department-depart, hardly-hard, and slipper-slip*). The behavioural priming effect for these materials was identical in size to the effect for morphologically transparent pairs like *teacher-teach*, consistent with the masked priming literature. The neural priming effect, however, was stronger in the visual word form area for the opaque and pseudostem pairs than for the transparent pairs, consistent with the view that activation at this level primarily reflects pre-lexical segmentation processes.^7^ Generally comparable findings, also linking morpho-orthographic effects to these inferior temporo-occipital areas, have recently been reported by Gold & Rastle (in press). Morpho-semantic effects, in contrast, seem to engage more anterior left middle temporal areas.

In auditory lexical processing, where information about morphemic components is delivered sequentially, there is extensive evidence that information carried by these components is extracted immediately it becomes available. This in turn links to growing evidence from neuropsychology and neuro-imaging that underlying neural systems are differentially sensitive to information carried by stems and by grammatical morphemes (e.g., Marslen-Wilson & Tyler, 2007; Tyler, Stamatakis, Post, Randall, & Marslen-Wilson, 2005). A critical issue for future research will be to link across these domains to explain how and why – not whether morphological analysis plays this key role in the functioning of the human language system.

## Appendix A: Stimulus Materials

**Table:**

Condition 1	prime	target	Condition 2	prime	target
−M−S +O	antique	ant	+M−S +O	archer	arch
−M−S +O	easel	ease	+M−S +O	blazer	blaze
−M−S +O	fluid	flu	+M−S +O	bracelet	brace
−M−S +O	curtain	curt	+M−S +O	choppy	chop
−M−S +O	ginger	gin	+M−S +O	coaster	coast
−M−S +O	harmony	harm	+M−S +O	cottage	cot
−M−S +O	grammar	gram	+M−S +O	crabby	crab
−M−S +O	fortune	fort	+M−S +O	grateful	grate
−M−S +O	napkin	nap	+M−S +O	muggy	mug
−M−S +O	nickel	nick	+M−S +O	petty	pet
−M−S +O	palace	pal	+M−S +O	porter	port
−M−S +O	pumpkin	pump	+M−S +O	whisker	whisk
−M−S +O	textile	text	+M−S +O	bloomer	bloom
−M−S +O	tinsel	tin	+M−S +O	corny	corn
−M−S +O	bandit	ban	+M−S +O	grubby	grub
−M−S +O	bunch	bun	+M−S +O	larder	lard
−M−S +O	chaplain	chap	+M−S +O	rafter	raft
−M−S +O	dollop	doll	+M−S +O	rattle	rat
−M−S +O	harpoon	harp	+M−S +O	rustic	rust
−M−S +O	humble	hum	+M−S +O	seedy	seed
−M−S +O	monkey	monk	+M−S +O	solvent	solve
−M−S +O	pillar	pill	+M−S +O	spanner	span
−M−S +O	scandal	scan	+M−S +0	sweater	sweat
−M−S +O	whimper	whim	+M−S +O	witness	wit
+ M midS + O	beefy	beef	−M mids −O	alloy	mix
+ M midS + O	bumper	bump	−M mids −O	blunt	dull
+ M midS + O	cracker	crack	−M mids −O	button	zip
+ M midS + O	folder	fold	−M mids −O	ceiling	roof
+ M midS + O	foxy	fox	−M mids −O	chisel	carve
+ M midS + O	frisky	frisk	−M mids −O	claw	hook
+ M midS + O	locker	lock	−M mids −O	cling	grab
+ M midS + O	prudent	prude	−M mids −O	confuse	stun
+ M midS + O	purely	pure	−M mids −O	dangle	tease
+ M midS + O	sneaker	sneak	−M mids −O	frond	fern
+ M midS + O	splinter	splint	−M mids −O	greed	envy
+ M midS + O	trailer	trail	−M mids −O	plaster	putty
+ M midS + O	urgent	urge	−M mids −O	attach	glue
+ M midS + O	barely	bare	−M mids −O	buzz	sing
+ M midS + O	boiler	boil	−M mids −O	choir	band
+ M midS + O	bowler	bowl	−M mids −O	deluge	flood
+ M midS + O	cheeky	cheek	−M mids −O	glimmer	spark
+ M midS + O	goatee	goat	−M mids −O	grievance	grudge
+ M midS + O	knotty	knot	−M mids −O	haggard	ugly
+ M midS + O	luggage	lug	−M mids −O	muddy	muck
+ M midS + O	lofty	loft	−M mids −O	rake	fork
+ M midS + O	solely	sole	−M mids −O	random	guess
+ M midS + O	stainless	stain	−M mids −O	squeeze	scratch
+ M midS + O	steamer	steam	−M mids −O	dentist	fang
+M +S +O	bravely	brave	−M + S −O	alley	lane
+M +S +O	calmness	calm	−M + S −O	arctic	polar
+M +S +O	cheerful	cheer	−M + S −O	rash	bang
+M +S +O	cruelly	cruel	−M + S −O	debt	owe
+M +S +O	densely	dense	−M + S −O	fabric	cloth
+M +S +O	dimly	dim	−M + S −O	jovial	merry
+M +S +O	drainage	drain	−M + S −O	lorry	truck
+M +S +O	foolish	fool	−M + S −O	pill	drug
+M +S +O	fussy	fuss	−M + S −O	pitcher	jug
+M +S +O	healer	heal	−M + S −O	rigid	stiff
+M +S +O	hunter	hunt	−M + S −O	scalpel	blade
+M +S +O	trashy	trash	−M + S −O	accuse	blame
+M +S +O	chilly	chill	−M + S −O	bunny	hare
+M +S +O	faithful	faith	−M + S −O	couch	sofa
+M +S +O	flashy	flash	−M + S −O	curve	bend
+M +S +O	graceful	grace	−M + S −O	frighten	scare
+M +S +O	herbal	herb	−M + S −O	mitten	glove
+M +S +O	heroic	hero	−M + S −O	mourn	sad
+M +S +O	lonely	lone	−M + S −O	shine	glow
+M +S +O	moisture	moist	−M + S −O	snort	sniff
+M +S +O	stormy	storm	−M + S −O	sorrow	grief
+M +S +O	thriller	thrill	−M + S −O	string	cord
+M +S +O	trickery	trick	−M + S −O	timid	shy
+M +S +O	wrecker	wreck	−M + S −O	devout	pious

## Footnotes

1 Later regression analyses confirmed that neither N, prime or target length, nor semantic relatedness, were significantly related to priming effects.

2 In the interests of completeness we also examined these issues at each SOA separately (with frequency effects again partialled out), with identical outcomes. At 36 ms we saw priming by morphology, *F*(2, 4241)=14.53, *p*<.0001, but no interaction of priming by morphology by semantics *F*(4, 4241)=0.45, *p*>.1; at 48 ms: priming by morphology *F*(2, 4276)=22.94, *p*<.0001; priming by morphology by semantics *F*(4, 4276)=0.18, *p*>.1; at 72 ms: priming by morphology *F*(2, 3875)=26.41, *p*<.0001; priming by morphology by semantics F(4, 3875)=0.18, *p*>.1.

3 In fact, when the items with phonemic differences between the pseudo-stem embedded in the prime and the pseudo-stem as target (e.g., *heaven-heave, galaxy-gala, signet-sign*, etc.) are removed from the critical Form and Opaque conditions in the Rastle et al. (2004) study, then priming levels for the remaining phonologically matched pairs seem statistically comparable across the two conditions.

4 These predictions also seem to hold for models where a three-way separation is made between a central level of semantic representation of lexical meaning, an intermediate lemma level, typically coding the syntactic properties of different lexemes, and a modality-specific level (orthographic or phonological) which represents the surface form of words and their constituent morphemes. From our perspective, the central semantic and the lemma levels group together, since they both operate at a modality-independent level of representation, and will both distinguish opaque from transparent complex forms. Words like *hardly and hard or archer* and *arch* will each need to have separate abstract lemma representations if they are to capture the necessary morpho-syntactic differences between them. Thus, whether priming is run off the central level, the lemma level, or through some interaction between them, opaque or pseudo-stem forms should show either no priming or reduced priming – in contrast to masked priming effects reflecting early segmentation of the orthographic input.

5 The labels ‘Prime’ and ‘Target’ need to be switched to get the correct values for the stem/ derived order.

6 The somewhat mixed pattern of semantic and phonological effects recently reported for overt tasks by Gonnerman, Seidenberg, and Andersen (2007) is likely to reflect individual variation in the representation of intermediate forms like ‘*lately/late’ or ‘midstream/stream*’, rather than the primary organisation of the underlying access system.

7 Devlin et al. (2004) themselves interpret these results quite differently, since they regard their pseudo-derived/semantically opaque condition (*department-depart, hardly-hard*) as a purely orthographic control for their morphologically related condition (*teacher-teach*). This does not seem tenable, given the ample evidence (including the results reported here) for the morphologically driven processing elicited by opaque and pseudo-derived pairs of exactly this type.
